# Environmental drivers of spatiotemporal foraging intensity in fruit bats and implications for Hendra virus ecology

**DOI:** 10.1038/s41598-018-27859-3

**Published:** 2018-06-22

**Authors:** John R. Giles, Peggy Eby, Hazel Parry, Alison J. Peel, Raina K. Plowright, David A. Westcott, Hamish McCallum

**Affiliations:** 1 Johns Hopkins University Bloomberg School of Public Health, Department of Epidemiology, Baltimore, MD USA; 2 Environmental Futures Research Institute, Griffith University, Brisbane, QLD Australia; 3 School of Biological, Earth, and Environmental Sciences, University of New South Wales, Sydney, NSW Australia; 4 CSIRO Health and Biosecurity, Brisbane, Queensland 4001 Australia; 5 Department of Microbiology and Immunology, Montana State University, Bozeman, MT USA; 6 CSIRO Land and Water, Atherton, Queensland 4883 Australia

## Abstract

In the Australian subtropics, flying-foxes (family Pteropididae) play a fundamental ecological role as forest pollinators. Flying-foxes are also reservoirs of the fatal zoonosis, Hendra virus. Understanding flying fox foraging ecology, particularly in agricultural areas during winter, is critical to determine their role in transmitting Hendra virus to horses and humans. We developed a spatiotemporal model of flying-fox foraging intensity based on foraging patterns of 37 grey-headed flying-foxes (*Pteropus poliocephalus*) using GPS tracking devices and boosted regression trees. We validated the model with independent population counts and summarized temporal patterns in terms of spatial resource concentration. We found that spatial resource concentration was highest in late-summer and lowest in winter, with lowest values in winter 2011, the same year an unprecedented cluster of spillover events occurred in Queensland and New South Wales. Spatial resource concentration was positively correlated with El Niño Southern Oscillation at 3–8 month time lags. Based on shared foraging traits with the primary reservoir of Hendra virus (*Pteropus alecto*), we used our results to develop hypotheses on how regional climatic history, eucalypt phenology, and foraging behaviour may contribute to the predominance of winter spillovers, and how these phenomena connote foraging habitat conservation as a public health intervention.

## Introduction

In 1930, frustration with the depredations of flying-foxes on commercial orchards led the Australian Government to commission an English researcher, Francis Ratcliffe, to undertake the first systematic investigation into the biology and ecology of pteropod bats [colloquially known as ‘flying-foxes’^1^]. Equipped with a motorbike, Francis Ratcliffe traveled thousands of miles across eastern Australia in an attempt to understand the nature and distribution of Australia’s four flying-fox species (*Pteropus scapulatus*, *P*. *conspiculatus*, *P*. *alecto*, and *P*. *poliocephalus*). At the time, virtually nothing was known about these pteropids except for anecdotal observations that they were numerous, volant, noisy, and often consumed agricultural fruit crops. Perceived as pests, management typically included destroying bats and roosts^2^. Motivated by these antagonistic interactions, Ratcliffe sought to understand flying-fox ecology in order to direct better-informed management strategies at that time. Our position is analogous to Ratcliffe’s 80 years ago, albeit with a few additional circumstances and tools. Competition for habitat between bats and humans continues, with the human footprint having engulfed considerable portions of bat foraging habitat^3^, leading to an increasing presence of flying-foxes in urban areas^4^. Human responses still include antagonistic control measures such as culling and dispersal, and ecologists are still working to understand the underlying ecology to direct better-informed conservation efforts.

In this context, understanding the foraging ecology of flying-foxes is critical. Hence, we focus on characterizing the observed foraging patterns of *P*. *poliocephalus* (colloquially known as the grey-headed flying-fox; hereafter GHFF). The GHFF is distributed along coastal habitat in eastern Australia, where it can form large colonies numbering into the hundreds of thousands^4,5^. They are highly mobile; populations disperse and coalesce quickly and dramatically in response to the availability of nectar and pollen produced by Eucalyptus trees (family Myrtaceae) in woodlands and open forests^6,7^. The best habitat for GHFF occurs in alluvial flats and riparian areas, where Eucalypt trees flower and produce nectar regularly during winter-spring; however, these areas have been largely cleared for agriculture^8^. The GHFF is listed as vulnerable in the IUCN Red List of Threatened Species due to population decline and winter-spring foraging habitat destruction. Although we focus on GHFF here, our study is timely given the negative impacts of anthropogenic change and poor management that affect a growing proportion of chiropterans around the world^9,10^.

GHFF foraging ecology is important because it plays a central ecological role in the health of eucalypt forests and in patterns of bat-borne disease dynamics, which are both major concerns for conservation strategies. Pteropids throughout the Old World pollinate numerous species of hardwood trees^11^. This role is especially crucial in Australia because eucalypt forests have considerable economic value as timber and they are primarily animal pollinated [i.e. mammals, birds, and insects^12^]. In contrast to this important ecosystem service, Australian flying-foxes are known reservoirs of Hendra virus. Hendra virus is transmitted from flying-foxes to horses when they forage in peri-urban landscapes. The virus is transmitted to humans after amplification within horses. Often lethal, Hendra virus infection causes severe neurological and respiratory disease in both horses and humans. flying-fox ecology drives the epidemiological dynamics of Hendra virus in bat populations and risk of spillover to horses^13^ through foraging behaviour and resulting population distribution^14^. Although, *P*. *alecto* (the black flying-fox; henceforth BFF) is thought to be the most significant source of viral excretion that leads to spillover, but foraging data is only available for GHFFs. GHFF exhibit seropositivity against Hendra virus^15^ and they share some foraging traits with BFFs such as the preference for nectar when it is available and reliance on anthropogenic food when nectar is not available^16,17^. Therefore, our results can be used to develop hypotheses about the links between eucalypt phenology, foraging behavior, and Hendra virus spillover, which is essential to assess conservation strategies in terms of both ecosystem and public health.

A major challenge to characterizing food resource availability for GHFF is the complex phenology of eucalypt forests. The GHFF preferentially seeks the highly-variable nectar and pollen of flowering eucalypts^16,18^. Many of these eucalypt species rely on subtle climate triggers to synchronize flowering among conspecifics^8,12,19^. The climate in eastern Australia can range from monsoon to extreme drought, depending on the inter-annual cycles of El Niño and La Niña, which are associated with sustained negative and positive values of the Southern Oscillation Index (SOI) respectively [collectively referred to as ENSO^20^]. As a result, eucalypt forests within the range of GHFF can exhibit variable spatiotemporal patterns in phenology depending on local species richness, climate, and environment.

When nectar resources across a landscape are poor (reduced abundance or patchy distribution), the GHFF adopts nomadic foraging behavior to search the landscape, visiting additional foraging areas to compensate for lower nectar availability^21^. In this manner, the GHFF exhibits a combined aggregation-dispersion foraging economy, where aggregation occurs during resource abundance, and dispersion occurs during resource shortage^22,23^. This occurs at multiple spatial and temporal scales. For example, an individual GHFF may visit a few highly productive foraging areas during some periods, but visit many less-productive foraging areas during other periods^21^. On a larger scale, many sub-populations of GHFF migrate long-distances when a high-quality flowering event occurs. This is observed when large numbers of GHFF converge in southern New South Wales for the prolific flowering events of *Corymbia maculata* (spotted gum), which occur every 3–4 years^5^. Such flexible population structure presents a considerable challenge to habitat conservation for flying-foxes because their foraging habitat and populations are constantly shifting over a large landscape^24^. Elucidating the spatiotemporal patterns of foraging activity in response to food resource phenology is, therefore, an important step to properly framing effective conservation efforts.

Previous research has shown that regional climate patterns in eastern Australia are connected to ENSO cycles^20^ and that flowering phenology in eucalypts depends on preceding climatic factors^8^. Therefore, we hypothesize that nectar foraging in flying-foxes is driven by eucalypt flowering events that respond to broader climatic patterns and we predict that models built with time-lagged spatial covariates representing these climatic patterns can characterize the spatial and temporal distribution of foraging areas. To explore this hypothesis, we present models and data of GHFF foraging intensity (defined as counts of foraging stops per unit area and time) around two roosting sites in southeastern Queensland. We use GPS collars and employ boosted regression trees to determine the environmental correlates of observed foraging events and summarize temporal changes in foraging intensity within the study area. Although the spatiotemporal variability in bat distribution and foraging habitat has been acknowledged as a major challenge to conservation^23–25^, to our knowledge, this is the first study to investigate the environmental drivers of flying-fox foraging activity and quantitatively describe its spatiotemporal patterns. We further explore spatial concentration of foraging intensity in comparison with independent population counts of *Pteropus poliocephalus*, the SOI, and the timing of Hendra virus spillover events.

## Results

**Figure 1:**
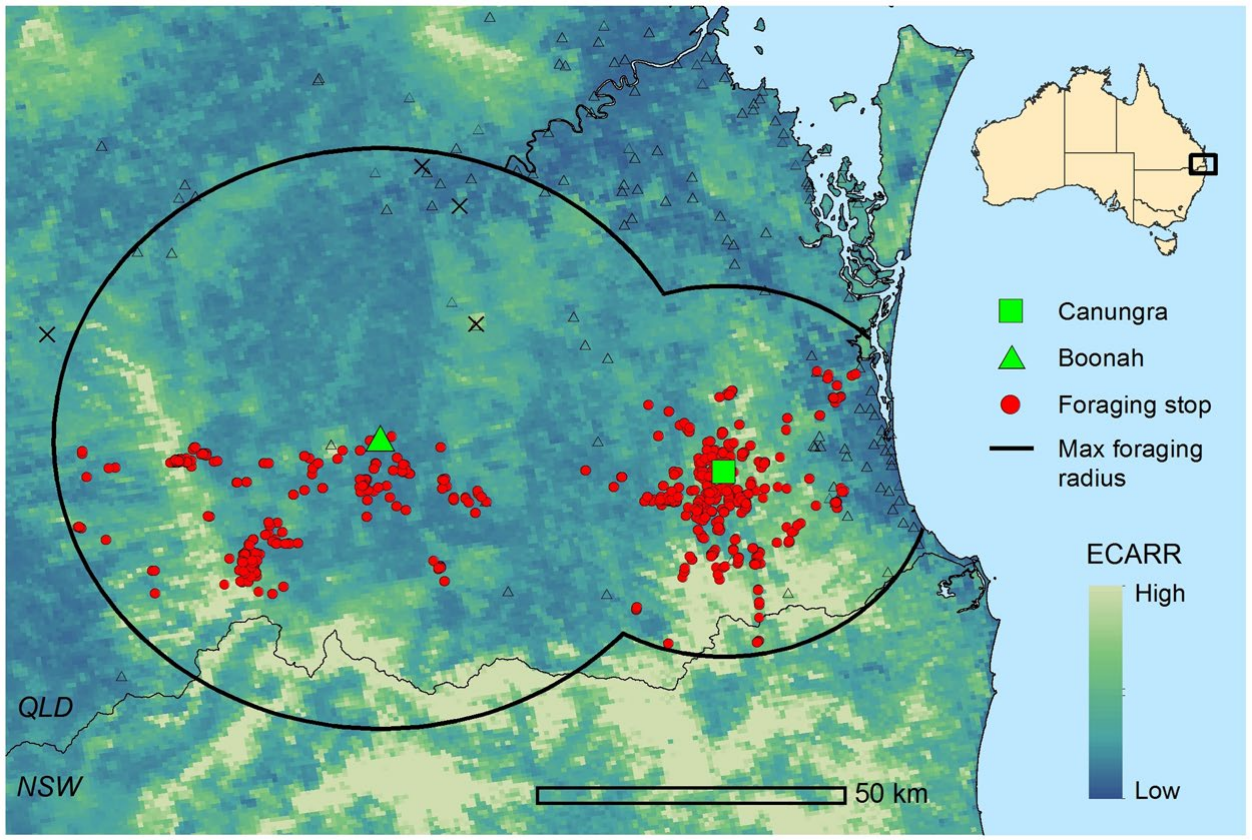
Study area of analysis, defined by the maximum observed foraging radius (46 km for Boonah and 30 km for Canungra), is plotted with unfiltered foraging areas (red points). Location of the roosts at Boonah and Canungra are shown as a green triangle and square respectively. The four black exes indicate foraging areas that were deemed outliers based on their proximity to different roosting sites. The black line represents the maximum foraging radius for *Pteropus poliocephalus* and the black triangles show locations of additional roosting sites. The background shows the distribution of the Eucalypt Chlorophyll *a* Reflectance Ratio (ECARR) which indicates areas with dense vegetation as light yellow and areas of less-dense vegetation as light blue. This map was generated using ESRI ArcGIS Desktop: Release 10 (www.esri.com).

**Figure 2:**
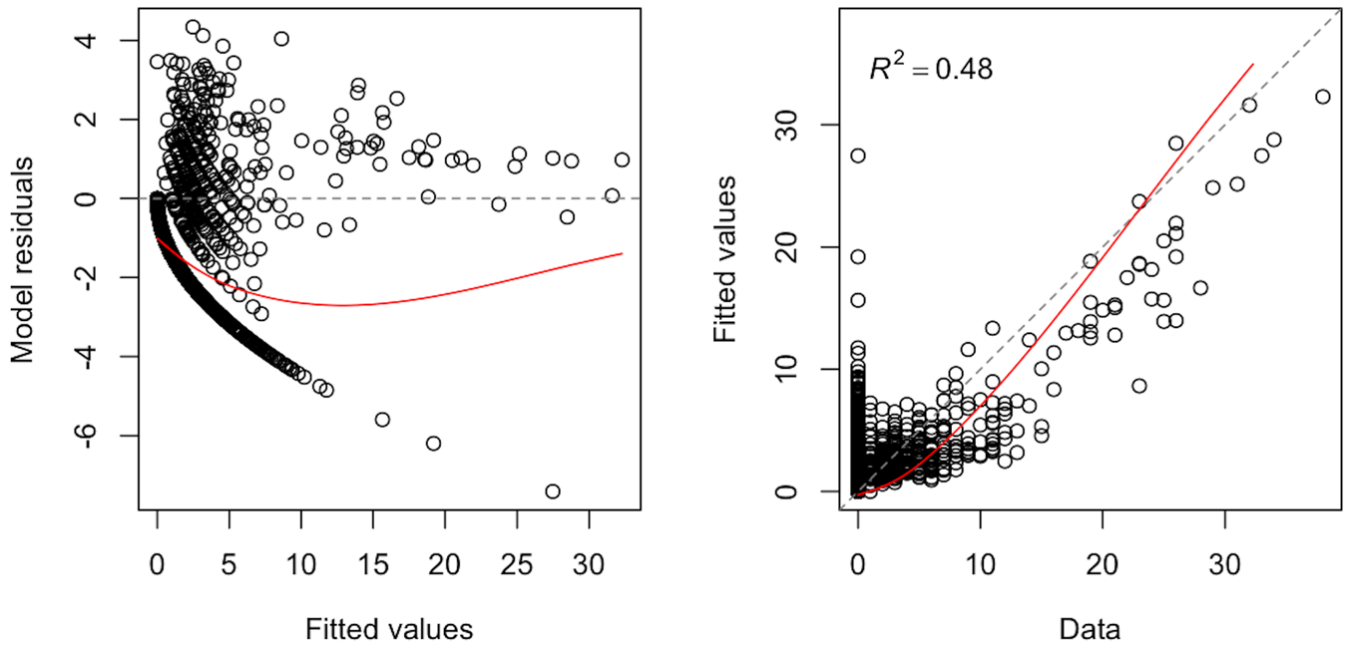
Standard model validation plots with fitted values versus model residuals on the left, and observed counts versus fitted values on the right. Both plots show adequate model fit with an *R*^2^ value of 0.48.

Data filtering and spatiotemporal aggregation of foraging areas used by our 37 GHFFs rendered 385 unique counts of foraging stops per 700 m grid cell within the study area from June 2013–Feb 2015 (Fig. 1). These count data represent counts of foraging stops per unit area and time, which we refer to as a measure of ‘foraging activity’ or ‘foraging intensity’. Model selection criteria based on performance within the 5-fold spatial cross validation structure returned 27 models giving the mean Poisson deviance and Mean Squared Error (MSE) (Table 1). The selected model (tree complexity (*tc*) = 9, learning rate (*lr*) = 0.001, bagging fraction (*bf*) = 0.6, number trees = 3400) had the lowest mean Poisson deviance (2.54) and MSE (4.11) while fitting >1000 trees. When the selected model was refitted with all data, it fitted 5100 trees. The final boosted regression tree model provided a valid fit to the data with evenly distributed residuals and an *R*^2^ value of 0.48 (Fig. 2).

**Table 1:** Performance of BRT models within the 5-fold gridded cross validation structure using different combinations of meta-parameters: tree complexity (*tc*), learning rate (*lr*), and bagging fraction (*bf*).

Tree complexity	Learning rate	Bagging fraction	No. trees	mean deviance	MSE
5	0.005	0.5	850	2.96	4.68
5	0.005	0.6	950	2.68	4.38
5	0.005	0.7	1200	2.62	4.35
5	0.001	0.5	4450	2.79	4.46
5	0.001	0.6	5000	2.74	4.42
5	0.001	0.7	5200	2.81	4.55
5	0.0005	0.5	8850	3.13	4.90
5	0.0005	0.6	10000	3.06	4.84
5	0.0005	0.7	9150	3.06	4.88
7	0.005	0.5	650	2.78	4.40
7	0.005	0.6	950	2.55	4.18
7	0.005	0.7	700	2.67	4.33
7	0.001	0.5	3200	2.74	4.36
7	0.001	0.6	4400	2.68	4.30
7	0.001	0.7	4000	2.61	4.24
7	0.0005	0.5	5600	2.95	4.60
7	0.0005	0.6	8200	2.72	4.34
7	0.0005	0.7	7200	2.70	4.34
9	0.005	0.5	350	3.03	4.68
9	0.005	0.6	600	2.68	4.28
9	0.005	0.7	650	2.51	4.09
9	0.001	0.5	2200	2.83	4.42
**9**	**0**.**001**	**0**.**6**	**3400**	**2**.**54**	**4**.**11**
9	0.001	0.7	3600	2.54	4.13
9	0.0005	0.5	4550	2.96	4.58
9	0.0005	0.6	6750	2.59	4.15
9	0.0005	0.7	7000	2.57	4.16

Performance measures reported are mean Poisson deviance and mean squared error (MSE) of the model across the 5-folds. The meta-parameter combination with the lowest mean deviance and MSE, and >1000 fitted trees is shown in bold.

**Figure 3:**
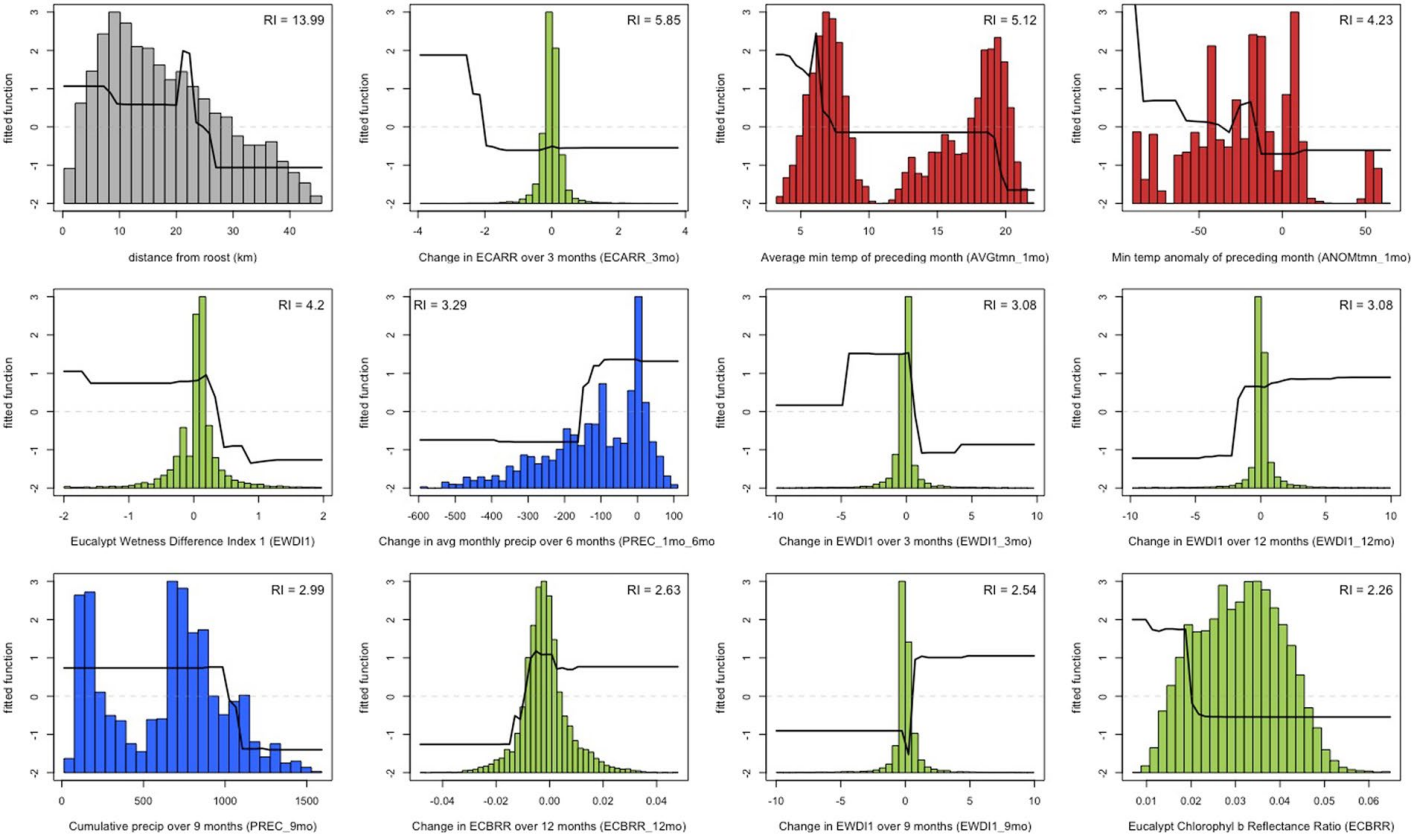
Partial dependence plots for 12 selected variables shown in order of importance. Scaled values on the x-axis show the standard deviation from the mean fitted response, and the range of each covariate is plotted on the x-axis. The black line shows the relative change in the fitted function over the range of each covariate. Histograms show the distribution of covariate values observed in the data, and the colors indicate the environmental variable type. Vegetation indices such as the Eucalypt Chlorophyll *a* Reflectance Ratio (ECARR), Eucalypt Chlorophyll *b* Reflectance Ratio (ECBRR), and Eucalypt Wetness Difference Index 1 (EWDI1) or plotted as green histograms. Temperature variables such as the average minimum temperature (AVGtmn) and minimum temperature anomaly (ANOMtmn) are plotted as red histograms, and precipitation variables such as the cumulative precipitation (PREC) and change in average monthly precipitation (PREC_1mo) are plotted as blue histograms. The percent relative influence (RI) is printed at the top of each plot.

Distance to roost was the most influential covariate in the model with a Relative Influence (RI) of 13.99% (Table 2). Other covariates with high RI include short-term changes in temperature and eucalypt vegetation indices such as ECARR_3mo (5.85%), AVGtmn_1mo (5.12%), ANOMtmn_1mo (4.23%), and EWDI1 (4.2%) (Tables 3 and 2). Partial dependence plots (Fig. 3) show that the fitted function is 1–2 standard deviations higher for foraging areas within 25 km of the home roost. Predicted foraging intensity in response to all vegetation indices (EWDI1, ECBRR, and ECARR; green plots in Fig. 3) was one standard deviation below the mean fitted response when the difference in the vegetation index over 9 or 12 month time-lags was negative, but the fitted response was one standard deviation above the mean when the indices were positive, indicating an increase in photosynthetic activity or tree canopy density over these longer time-lags. Over shorter time intervals (3-month time-lags and the current month), the fitted response was up to 2 standard deviations above the mean when the difference in vegetation indices was negative, indicating that decreases in vegetation indices over the preceding 1–3 months are associated with a higher level of foraging intensity.

**Table 2:** The 24 most influential variables as determined by the boosted regression tree model, shown in order of relative influence.

Predictor	Description	Relative influence (%)
**DFR**	Distance from roost	13.99
**ECARR_3mo**	Change in ECARR over 3 months	5.85
**AVGtmn_1mo**	Average minimum temperature of preceding month	5.12
**ANOMtmn_1mo**	Cumulative minimum temperature anomaly over preceding month	4.23
**EWDI1**	Eucalypt Wetness Difference Index 1	4.20
ECBRR_6mo	Change in ECBRR over 6 months	3.70
**PREC_1mo_6mo**	Change in PREC_1mo over 6 months	3.29
**EWDI1_3mo**	Change in EWDI1 over 3 months	3.08
**EWDI1_12mo**	Change in EWDI1 over 12 months	3.08
**PREC_9mo**	Cumulative precipitation of the preceding 9 months	2.99
PREC_12mo	Cumulative precipitation of the preceding 12 months	2.92
EWDI2_1mo	Change in EWDI2 over 1 month	2.70
**ECBRR_12mo**	Change in ECBRR over 12 months	2.63
**EWDI1_9mo**	Change in EWDI1 over 9 months	2.54
ANOMtmx_9mo	Cumulative maximum temperature anomaly over preceding 9 months	2.44
ECARR_12mo	Change in ECARR over 12 months	2.40
**ECBRR**	Eucalypt Chlorophyll *b* Reflectance Ratio	2.26
ECBRR_9mo	Change in ECBRR over 9 months	2.12
EWDI2_6mo	Change in EWDI2 over 6 months	2.10
AVGtmx_1mo	Average maximum temperature of preceding month	2.10
PREC_1mo	Cumulative precipitation of the preceding month	1.96
ANOMtmn_3mo	Cumulative minimum temperature anomaly over preceding 3 months	1.84
PREC_1mo_12mo	Change in PREC_1mo over 12 months	1.57
ECARR_9mo	Change in ECARR over 9 months	1.57

Predictors plotted in Fig. 3 are indicated in bold.

**Table 3:** A complete list of all environmental variables explored in the model.

Name	Description	Formula
ECARR	Eucalypt Chlorophyll *a* Reflectance Ratio	0.0161 × [Band2/(Band4 × Band1)]^0.7784^
ECARR_3mo	Change in ECARR over 3 months	ECARR_*t*_ − ECARR_*t*−3*months*_
ECARR_6mo	Change in ECARR over 6 months	ECARR_*t*_ − ECARR_*t*−6*months*_
ECARR_9mo	Change in ECARR over 9 months	ECARR_*t*_ − ECARR_*t*−9*months*_
ECARR_12mo	Change in ECARR over 12 months	ECARR_*t*_ − ECARR_*t*−12*months*_
ECBRR	Eucalypt Chlorophyll *b* Reflectance Ratio	0.0337 × (Band1/Band4)^1.8695^
ECBRR_3mo	Change in ECBRR over 3 months	ECBRR_*t*_ − ECBRR_*t*−3*months*_
ECBRR_6mo	Change in ECBRR over 6 months	ECBRR_*t*_ − ECBRR_*t*−6*months*_
ECBRR_9mo	Change in ECBRR over 9 months	ECBRR_*t*_ − ECBRR_*t*−9*months*_
ECBRR_12mo	Change in ECBRR over 12 months	ECBRR_*t*_ − ECBRR_*t*−12*months*_
EWDI1	Eucalypt Wetness Difference Index 1	0.08 × [(Band2 − Band7)/(Band2 − Band6)] − 0.052
EWDI1_3mo	Change in EWDI1 over 3 months	EWDI1_*t*_ − EWDI1_*t*−3*months*_
EWDI1_6mo	Change in EWDI1 over 6 months	EWDI1_*t*_ − EWDI1_*t*−6*months*_
EWDI1_9mo	Change in EWDI1 over 9 months	EWDI1_*t*_ − EWDI1_*t*−9*months*_
EWDI1_12mo	Change in EWDI1 over 12 months	EWDI1_*t*_ − EWDI1_*t*−12*months*_
EWDI2	Eucalypt Wetness Difference Index 2	0.045 × [(Band2 − Band6)/(Band2 − Band7)] − 0.014
EWDI2_3mo	Change in EWDI2 over 3 months	EWDI2_*t*_ − EWDI2_*t*−3*months*_
EWDI2_6mo	Change in EWDI2 over 6 months	EWDI2_*t*_ − EWDI2_*t*−6*months*_
EWDI2_9mo	Change in EWDI2 over 9 months	EWDI2_*t*_ − EWDI2_*t*−9*months*_
EWDI2_12mo	Change in EWDI2 over 12 months	EWDI2_*t*_ − EWDI2_*t*−12*months*_
ANOMtmn_1mo	Cumulative minimum temperature anomaly over preceding month	
ANOMtmn_3mo	Cumulative minimum temperature anomaly over preceding 3 months	
ANOMtmn_6mo	Cumulative minimum temperature anomaly over preceding 6 months	
ANOMtmn_9mo	Cumulative minimum temperature anomaly over preceding 9 months	
ANOMtmn_12mo	Cumulative minimum temperature anomaly over preceding 12 months	
ANOMtmx_1mo	Cumulative maximum temperature anomaly over preceding month	
ANOMtmx_3mo	Cumulative maximum temperature anomaly over preceding 3 months	
ANOMtmx_6mo	Cumulative maximum temperature anomaly over preceding 6 months	
ANOMtmx_9mo	Cumulative maximum temperature anomaly over preceding 9 months	
ANOMtmx_12mo	Cumulative maximum temperature anomaly over preceding 12 months	
AVGtmn_1mo	Average minimum temperature of preceding month	
AVGtmx_1mo	Average maximum temperature of preceding month	
PREC_1mo	Cumulative precipitation of the preceding month	
PREC_3mo	Cumulative precipitation of the preceding 3 months	
PREC_6mo	Cumulative precipitation of the preceding 6 months	
PREC_9mo	Cumulative precipitation of the preceding 9 months	
PREC_12mo	Cumulative precipitation of the preceding 12 months	
PREC_1mo_3mo	Change in PREC_1mo over 3 months	PREC_1mo_*t*_ − PREC_1mo_*t*−3*months*_
PREC_1mo_6mo	Change in PREC_1mo over 6 months	PREC_1mo_*t*_ − PREC_1mo_*t*−6*months*_
PREC_1mo_9mo	Change in PREC_1mo over 9 months	PREC_1mo_*t*_ − PREC_1mo_*t*−9*months*_
PREC_1mo_12mo	Change in PREC_1mo over 12 months	PREC_1mo_*t*_ − PREC_1mo_*t*−12*months*_

**Figure 4:**
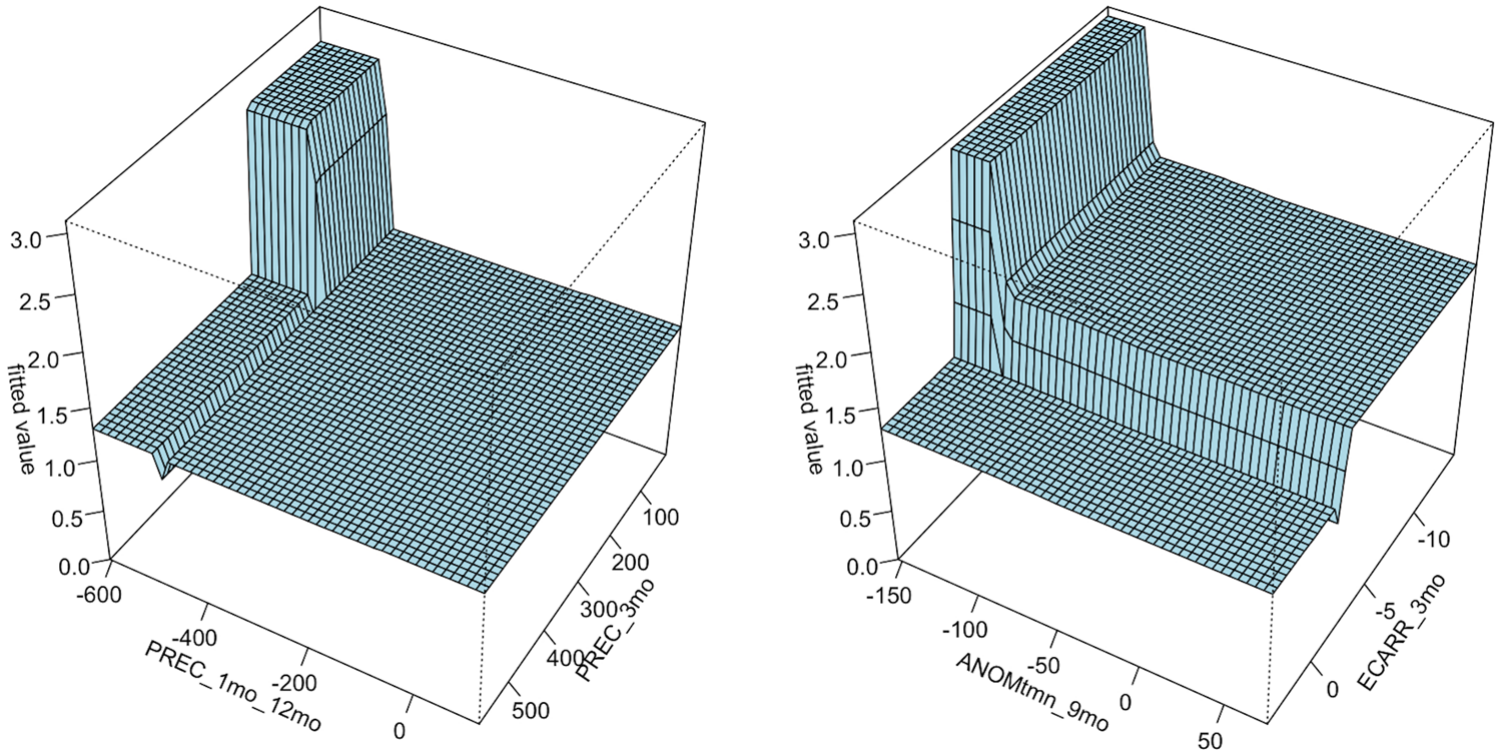
The two most influential interactions fitted by the boosted regression tree model. Left: Fitted values are plotted as a function of the change in monthly precipitation over 12 months (PREC_1mo_12mo) and the cumulative precipitation over the preceding 3 months (PREC_3mo). The plot shows higher levels of foraging when decreases compared with the previous year and cumulative amounts of rainfall remain below 200 mm in the preceding 3 months. Right: Fitted values plotted as a function of the minimum temperature anomaly of the preceding 9 months (ANOMtmn_9mo) and the change in the Eucalypt Chlorophyll *a* Reflectance Ratio over 3 months (ECARR_3mo). Here, highest fitted values correspond with low minimum temperature in the past 9 months and decrease in photosynthetic productivity over 3 months.

In terms of climatic conditions, temperature variables (red plots in Fig. 3) show that predicted foraging intensity falls dramatically when average minimum temperature (AVGtmn_1mo) rises above 20 °C, but it increases when the minimum temperature anomaly (ANOMtmn_1mo) is negative (representing an especially cool month). Precipitation variables (blue plots in Fig. 3) show that severe decreases in precipitation over 6 months (PREC_1mo_6mo) are not associated with foraging activity, and high values (>1000 mm) of cumulative rainfall over the preceding 9 months (PREC_9mo) coincide with a dramatic decrease in predicted foraging activity. Interactions between environmental variables provide further description of the fitted function. Specifically, the highest levels of foraging activity are fitted when both of the following conditions are met: 1) monthly precipitation is significantly less than the prior year and <200 mm of rainfall in the preceding 3 months, and 2) the minimum temperature anomaly of the preceding 9 months is low and coincides with a decrease in ECARR over 3 months (Fig. 4). In general, the partial dependence plots and interactions indicate that foraging activity is more intense when summer and autumn conditions are cooler than average with relatively reduced precipitation compared with previous months, and photosynthetic productivity in eucalypts diminishes. This emphasizes the importance of past conditions (3–12 months previous) that are warmer and wetter, suggesting that a moderate *transition* from warm/wet to cool/dry is a primary environmental driver of eucalypt flowering and flying-fox foraging intensity within our study area.

**Figure 5:**
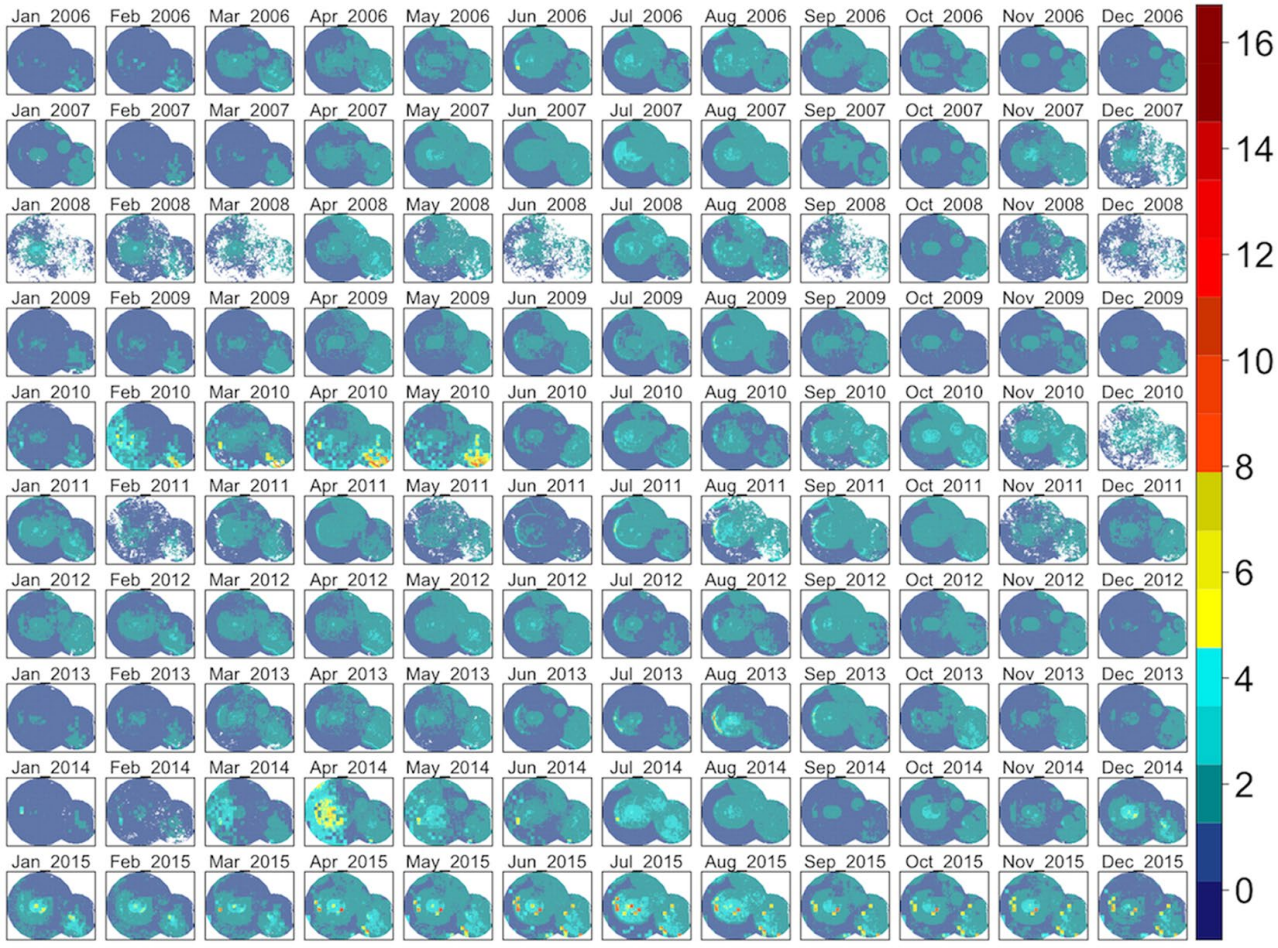
Monthly spatial predictions of the final boosted regression tree model within the maximum observed foraging radius of *Pteropus poliocephalus* around the two study roosts in southeastern Queensland (46 km for Boonah and 30 km for Canungra) from 2005–2014. Predicted number of foraging stops is shown from dark blue (*Y* = 0), to red (*Y* = 16).

**Figure 6:**
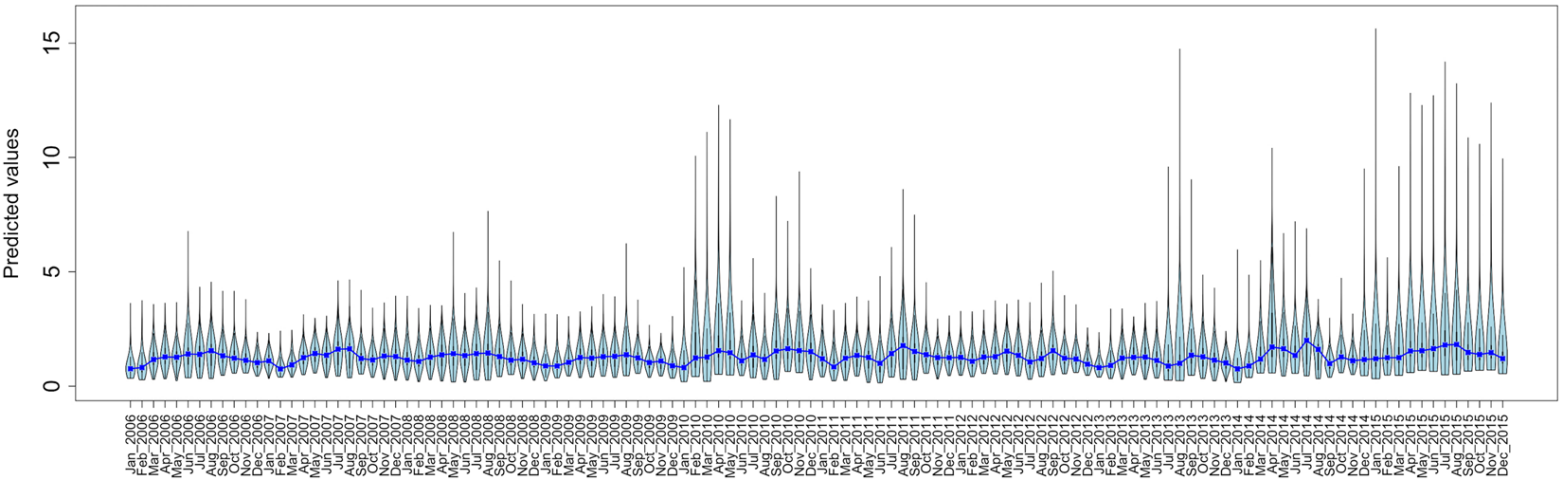
A time series of the final boosted regression tree model within the maximum observed foraging radius of *Pteropus poliocephalus* around the two study roosts in southeastern Queensland (46 km for Boonah and 30 km for Canungra) from January 2006–December 2015. The violin plots show the distribution of all cell values within the study area and the blue line shows the change of the median value over time.

**Figure 7:**
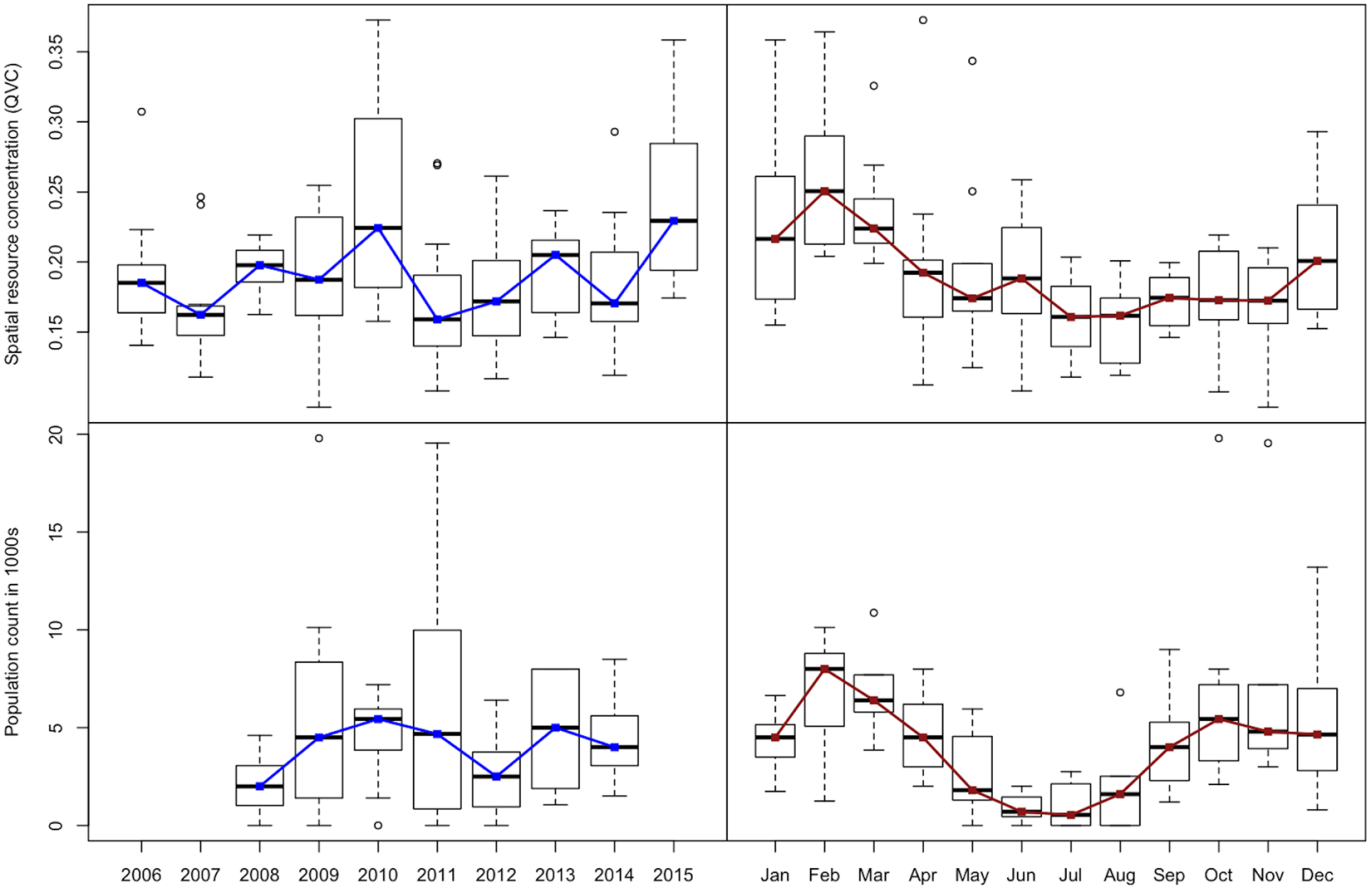
Annual and seasonal trends in model predictions are plotted against the quartile variation coefficient (QVC), which is a measure of spatial resource concentration (top panels) and independent data of population census counts for the grey headed flying-fox at the Canungra roosting site (bottom panels).

When the final model is projected across the study area, foraging intensity exhibits a patchy spatial distribution that is qualitatively consistent with typical heterogeneous patterns of eucalypt flowering (Fig. 5). Similarly, when plotted as a time series and aggregated by year (Figs 6 and 7 respectively), model predictions also display interannual variability. Model predictions summarized by month for foraging areas within 30 km of the Canungra roost show that the measure of spatial resource concentration (QVC) is typically higher in mid-summer and early autumn (Dec–Mar; Fig. 7). Beyond seasonal patterns, foraging areas around Canungra exhibit some interannual variation also, with mid-summer months in 2010 and 2015 being particularly high (9). We found that independent monthly population counts of *Pteropus poliocephalus* observed at Canungra from 2008–2014 were significantly correlated with predicted values of monthly foraging intensity (*r* = 0.74, 95% CI = 0.28, 0.92, p = 0.006, n = 12; Fig. 7), giving strong validation that the fitted model adequately captures seasonal trends in foraging intensity. Comparison of the model to population counts across years did not show a significant correlation (*r* = 0.31, 95% CI = −0.58, 0.86, p = 0.5, n = 5). However, from 2008–2014, the model predicts the highest foraging intensity in 2010 and 2013, the same years in which median population counts are highest. More broadly, time-lagged cross correlation reveals that model predictions are significant and positively correlated with the SOI values 3–8 months previous (n = 120; Fig. 10), as seen by slightly lagged alignment of peaks in predicted foraging intensity with peaks in SOI values (Fig. 11).

**Figure 8:**
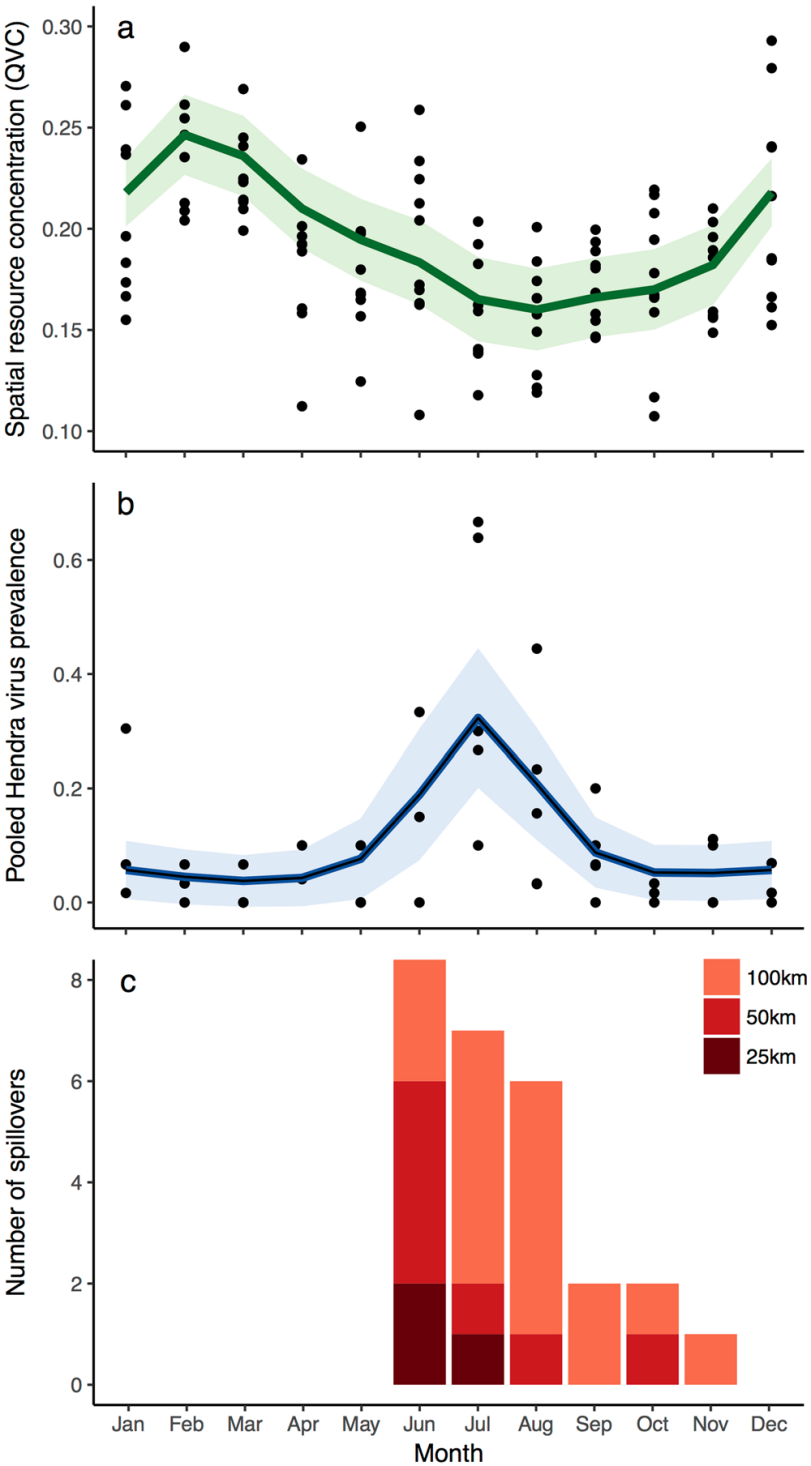
Seasonal trends in (**a**) spatial resource concentration as quantified by the quartile variation coefficient (QVC) within 50 km of both Boonah and Canungra roosts, (**b**) Hendra virus prevalence at the Boonah roost from July 2011–June 2014 (prevalence calculated from pooled under-roost urine samples^26^), and (**c**) the total number of spillover events within 25, 50, and 100 km of the both Boonah and Canungra roosts. Lines in (**a**) and (**b**) represent fitted Generalized Additive Models with cubic cyclic spline smoothing terms and their 95% confidence intervals.

To explore our model of spatial resource concentration in the context of Hendra virus ecology, we plotted our model results along with existing data of Hendra virus prevalence at the Boonah roosting site^26^ and spillover events in horses^27^ that allowed comparison of seasonal trends among the three phenomena (Fig. 8). The Generalized Additive Model (GAM) that we fit to QVC values (n = 120, P-value of smooth term (month) = 3.7 × 10^−9^, deviance explained = 31.4%) shows that the QVC is highest in late-summer and lowest in winter (Fig. 8a). The GAM fitted to Hendra virus prevalence data (n = 40, P-value of smooth term (month) = 1.2 × 10^−4^, deviance explained = 45.4%) showed a peak in prevalence during winter (Jun–Aug; Fig. 8b). When we plotted the total counts of Hendra virus spillover incidents, all three distance thresholds (25, 50, and 100 km) showed the same seasonal pattern with the highest count occurring in June with decreasing counts in subsequent months. Within 25 km of our study roost sites there have been a total of: 3 spillover incidents which have occurred within 25 km during Jun–Jul, 7 spillover incidents within 50 km during Jun–Oct, and 20 spillover incidents within 100 km during Jun–Nov (Fig. 8c).

## Discussion

We developed a spatiotemporal model of flying-fox foraging intensity that is validated by independent data of seasonal bat population counts and provides a quantitative link between bat foraging dynamics and cycles of the ENSO. Specifically, increased rainfall and minimum temperatures (typical of La Niña events and favorable for the growth of eucalypts) in preceding seasons drive flowering and spatially concentrated foraging intensity in the following autumn. We also found that in the same region as our study area, both observed prevalence of Hendra virus in bat populations, and occurrence of spillover to horses, align with seasonal and annual periods when our model indicates a low concentration of nectar-based resources (Fig. 8). Nectar scarcity may drive bats to feed in proximity to horses or may modulate bat immune status leading to virus shedding^13,14^. Further, future climate changes will likely alter climatic conditions during important growth phases of eucalypts^28^, affecting the spatiotemporal distribution of nectar-based foraging resources for flying-foxes, which could have implications for both Hendra virus ecological dynamics and strategies for habitat conservation.

Previous research into the movement ecology of bats has shown important aspects of mobility and habitat preference. Telemetry and GPS tracking studies have looked at the scale of movement^29–33^, habitat preference^34,35^, and seasonal differences in foraging behavior between wet and dry seasons^36^ in several species of flying-fox, which emphasize that mobility and generalist foraging behavior allow population redistribution in response to food resources. Previous studies have also modeled the spatial extent of bat habitat using roost locations^37–39^. However, models based on roost locations are limited when describing foraging habitat. Giles *et al*.^40^ modeled population occupancy and abundance at day roosts with proxies of eucalypt flowering. They addressed the discrepancy between roosting and foraging habitat by taking a spatially weighted sample of model predictors within areas known to contain diet species of bats. However, robust models of foraging habitat require explicit data of occupied foraging areas. The high-resolution tracking data collected in Westcott *et al*.^4^ allowed us to identify these foraging areas with high confidence. When combined with spatial proxy variables of eucalypt phenology from Giles *et al*.^40^ we built a model of foraging intensity at the regional scale, which serves as a starting point for developing landscape scale models.

Environmental variables that predict bat foraging intensity are consistent with known climatic triggers of eucalypt flowering. We found that, in our study area, high predicted values of foraging intensity were associated with long-term increases (9–12 months) but short-term decreases (1–3 months) in eucalypt vegetation indices (green plots in Fig. 3). This suggests that there is a sequence of key seasonal time periods prior to foraging when phenological changes in eucalypts occur (e.g. growth phase, bud production, anthesis), leading to flowering and nectar availability in the following months. We propose then, that a slowing of vegetative growth in the eucalypt canopy is an important precursor to foraging activity, which is consistent with the reproductive phenology of eucalypts. Generally, growth and flowering are separate processes, where vegetative growth is suppressed in favor of bud development and floral initiation^41,42^. In many species, these changes from one phenological stage to the next (i.e. growth to flowering) are initiated by cooler temperatures [as documented for *Eucalyptus regnans*, *E*. *maculata*, and *E*. *acmenoides*^43,44^].

In terms of climate, we found that foraging intensity was higher during periods that are cooler and drier than average, especially if they are preceded by conditions that are warmer and wetter than average. Interaction plots (Fig. 4) show that flowering and foraging is favored when precipitation slows over the preceding year, indicating that previous rainfall followed by a transition from wet to dry is important. Similarly, Law *et al*.^8^ observed that, for 20 species of eucalypts in northern New South Wales, the greatest flowering in a 10 year period occurred 9 months after an above average rainfall event. Further, it is interesting that partial dependence plots (blue plots in Fig. 3) indicate that in the region of our study area, changes in precipitation must be relatively mild, and that extreme transitions (e.g. an exceedingly wet period followed by an average one, or an average period followed by a drought) may not be amenable to flowering. We speculate that during wet periods, eucalypt forests may forgo flowering in favor of continued growth, and during a drought, dry conditions inhibit flowering and nectar production due to moisture stress.

When considering temperature, we found that flowering and foraging is associated with low minimum temperatures (below 19 °C) in the preceding month (red plots in Fig. 3). Likewise, Law *et al*.^8^ showed that prior cool temperatures were the most consistent climatic trigger for flowering in 9 of 20 eucalypt species in northern New South Wales. In more southern species, Specht and Brouwer^44^ note that in *E*. *regnans* the growth phase is halted when mean temperatures drop below 16–18 °C, followed by flowering [typically in autumn for this species^43^], and Moncur^45^ demonstrated floral induction in *E*. *lansdowneana* below 10–15 °C. Our model results complement previous studies on flowering phenology within the context of flying-fox foraging ecology, showing that cool temperatures are an important seasonal climatic trigger determining the timing of flowering in eucalypts, which leads to nectar-based resources for flying-foxes.

Beyond seasonal climatic drivers, we observed a positive correlation between the spatial concentration of nectar foraging and values of the SOI at a temporal lag of 3–8 months (Fig. 10), indicating that previous climatic conditions associated with La Niña have a cumulative effect on flowering phenology and resulting foraging by flying-foxes. It has been known for some time that the state of the ENSO can predict spring/summer rainfall for eastern Australia^20^, so the observed correlation is not entirely surprising. SOI values above zero are associated with La Niña cycles, where increased ocean surface temperatures increase cloud cover, humidity, rainfall, and minimum temperatures for southeastern Queensland^46^. Some species of eucalypt may invest more energy into the growth phase during the amenable conditions of La Niña, until drier and cooler conditions of a neutral phase trigger flowering. However, it should be noted that each species of eucalypt has a unique seasonal schedule for growth and flowering phenology that is influenced by both photoperiod and time-lagged climatic effects^47^. Therefore, physiological responses to the broader climatic conditions brought by La Niña may influence eucalypts within a region by means of the Moran effect^48^, causing periodic deviations from strict seasonal dynamics. We hypothesize, accordingly, that the timing of ENSO cycle changes (i.e. La Niña to neutral phase) is a general driver of the notorious inter-annual periodicity of eucalypt growth and flowering phenology, and the erratic patterns of redistribution characteristic of flying-fox populations.

Seasonal and annual trends in model predictions indicate that nectar-based foraging for flying-foxes has important implications for Hendra virus disease ecology. As shown in Fig. 8a,b, seasonal trends in the model predict peaks in spatial resource concentration in late-summer, followed by lower values in autumn/winter. Coincidentally, Field *et al*.^26^ and Paez *et al*.^49^ show that peak Hendra virus prevalence in bat populations also occurs in the winter, which may indicate epidemiological drivers of Hendra virus prevalence in bat roosts in response to resource scarcity. It should be noted that our model of resource distribution is based on the foraging patterns of GHFF whereas BFF is thought to be the primary driver of Hendra virus transmission in bat populations and excretion of the virus into the environment^15^. However, Paez *et al*.^49^ found that measures of climate in preceding seasons (similar to those used in our model) in combination with the number of BFF could predict peaks in Hendra virus prevalence. Specifically, they found that when hot and dry conditions in the previous spring/summer coincide with larger numbers of BFF, peaks in Hendra virus are higher and occur earlier in the winter season. Based on our model and its correlation with the SOI, warmer and drier conditions in the spring/summer (typical of the El Niño phase of ENSO), appear connected with both reduced flowering and foraging in the following autumn/winter, and larger peaks in Hendra virus prevalence in the winter season. Following Plowright *et al*.^14^, a period of resource scarcity could impact viral prevalence in bat populations through a decreased allocation of energy to immune defenses. Resource scarcity could also increase contact rates and connectivity between local roosts due to more dispersed foraging behavior in the region, thus affecting viral dynamics.

**Figure 9:**
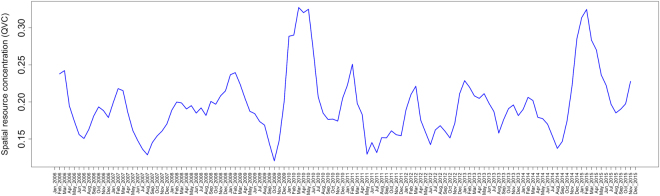
A univariate time series of the quartile variation coefficient (QVC), which is a measure of spatial resource concentration, was calculated from spatial model predictions within the maximum foraging radius of only the Canungra roost (30 km).

Spillover events from flying-foxes to horses in the region follow a similar pattern with winter occurrence (Fig. 8c)^13^. Plowright *et al*.^13^ also note that there was an unprecedented cluster of spillover events in the winter of 2011 in southern Queensland and northern New South Wales, a year in which our model predicts the lowest concentration of nectar-based resources around Canungra (Figs 7 and 9). We, therefore, hypothesize that periods of a low spatial concentration of nectar resources result in dispersed foraging behavior among resident GHFF and BFF individuals, which increases the number of bats foraging in horse paddocks. In this manner, the type of population distribution across the landscape (e.g. aggregated during resource plenty or dispersed during resource scarcity) determines where the virus can be excreted into the environment, providing the first enabling factor for spillover to horses^50^.

**Figure 10:**
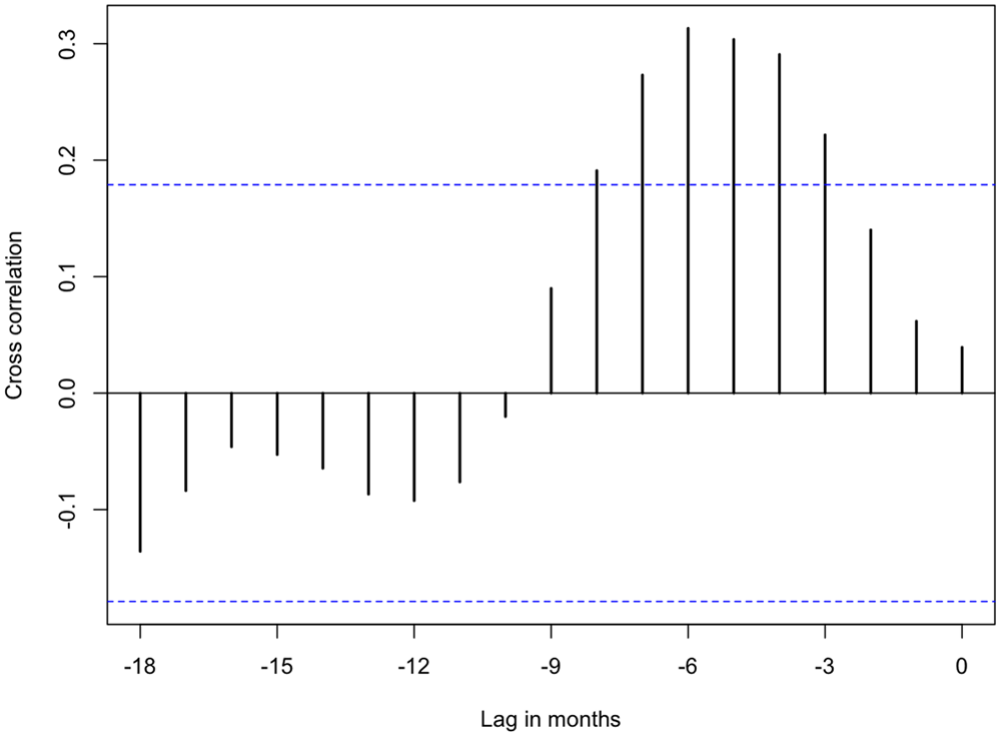
The cross-correlation function showing the time-lagged correlation between the quartile variation coefficient (QVC). The QVC was calculated from spatial model predictions within the maximum observed foraging radius of *Pteropus poliocephalus* around the two study roosts in southeastern Queensland (46 km for Boonah and 30 km for Canungra) and the a 3-month moving average of the Southern Oscillation Index (SOI). Dashed blue lines indicate the cutoff for significance for the cross-correlation coefficient. Time series of the QVC an SOI are plotted in Fig. 11.

**Figure 11:**
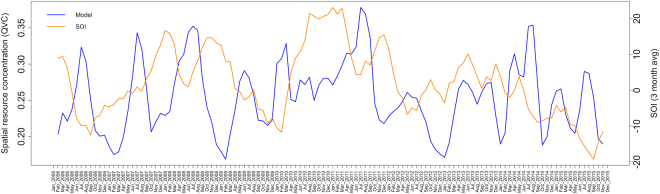
The quartile variation coefficient (QVC), which is a measure of spatial resource concentration, was calculated from spatial model predictions within ~50 km of both Boonah and Canungra roosts and plotted with a 3-month moving average of the Southern Oscillation Index (SOI) from January 2006–December 2015. The QVC values are plotted as the blue line and the SOI values are plotted as the orange line.

The role of foraging behavior in bat population distribution and Hendra virus ecology emphasizes the importance of coherent management actions which preserve or enhance flying-fox foraging habitat. This is especially timely because a 2013 amendment to the Queensland Vegetation Management Act 1999 loosened regulations on remnant and old growth forest clearing. A subsequent resurgence of vegetation clearing [395,000 hectares per year in 2015–16^51^], put Queensland on a trajectory to return to the clearing rates of the 1990s when it was labeled a world deforestation ‘hotspot’^52,53^. In response, the Queensland government reinstituted legislation this year to protect remnant vegetation^54^, which is a promising step to reduce future clearing rates. However, the impacts of clearing on bat populations and risk of spillover may not be observed until subsequent foraging bottlenecks occur due to annual or inter-annual cycles in climate. For instance, cleared foraging areas for bats may not cause significant changes in nectar resource availability and foraging behavior until the next El Niño phase when warmer and dryer spring/summer conditions cause poor flowering in the following autumn. The time-lagged effects of deforestation and habitat fragmentation, therefore, present significant challenges when studying the ecological impacts of landscape change. Hence, habitat conservation strategies should employ analyses on the dynamic changes in flowering and foraging over time to identify areas that: 1) offer consistent winter flowering species, and 2) contain species that produce large amounts of nectar on an inter-annual basis, and seek to preserve these areas, or even establish new areas to plant important diet species. Such a conservation strategy targets both temporal and spatial gaps in nectar-based resources^24^.

Our model is subject to three important limitations inherent in the data that must be considered. First, the data that we used to define foraging areas of bats came from 37 individuals roosting at Boonah and Canungra between June 2013–February 2015. Although we have confidence in our definition of foraging areas, we must assume that the areas used by our 37 individuals are representative of a larger population. Second, there is likely spatial autocorrelation in the foraging area data (even after we clustered foraging stops into areas). We, therefore, designed a modeling approach that reduces this influence by aggregating foraging areas to more coarse spatial and temporal scales, utilizing the ‘bagging’ technique within the BRT algorithm, including distance from the home roost as a predictor, and by determining model meta-parameters using a 5-fold spatial cross validation structure. Even with appropriate precautions, it is difficult to completely remove the confounding effects of autocorrelation in the data. Third, there is heterogeneous species richness in eucalypts within the study area, which may lead to diverse responses to climate. There is likely variability in foraging dynamics that is not included in our model because the model predictions are summarized over coarse spatial and temporal scales in order to draw broader inferences. This is a trade-off that must be made in order to describe the *general* environmental drivers of flowering and bat foraging within the region of our two study roosts over time. Therefore, our conclusions are relative to observed foraging activity within our study area, and differences in eucalypt species richness and regional climate regimes may lead to different results in other regions.

So then, are we any further along than Ratcliffe in implementing ‘better-informed conservation’? Recent dispersal actions^55^ indicate that, in terms of implementation, the answer is no. However, we are making progress in understanding the dynamics of bat population distribution, which is difficult given the spatial scale at which they operate. Here we showed that the climatic responses of eucalypt phenology drive bat foraging, which has important implications for bat population distribution, Hendra virus ecology, and conservation strategy. This provides additional ecological context to previous work, and connects ecosystem phenology to broader climatic drivers such as the ENSO, allowing speculation about the future impacts of climate changes. For example, under future climate predictions, the frequency of El Niño cycles are expected to increase, with more irregular timing^56^. Based on our data and model, this would result in less reliable temperatures and rainfall in spring/summer seasons, contributing to a decrease in the spatial concentration of flowering and foraging in our study area. Therefore, future research that takes the concepts presented here, and applies them to the landscape scale could produce a useful spatiotemporal map defining priority conservation areas that provide sufficient foraging habitat over time. Additionally, mechanistic models of bat distribution that include this information would help to understand the spatiotemporal distribution of bat populations in a human-dominated landscape, and the potential for food resource phenology to drive Hendra virus ecological dynamics.

## Methods

### Environmental data

We developed a suite of environmental data layers for the rectangular extent encompassing the maximum foraging radii of two study roost sites (Boonah and Canungra; Fig. 1) from 2006–2015 at 8-day intervals (spatial resolution of 700 m in Albers Equal Area projection). These include Eucalypt-based vegetation indices, average monthly precipitation, cumulative precipitation, average monthly temperature extrema, and temperature anomaly. The Eucalypt-based vegetation indices are spatial adaptations of laboratory measurements from Datt^57,58^, which represent chlorophyll and water content of Eucalypt leaves. For a more extensive discussion of the indices and climate variables, see Giles *et al*.^40^. Given the significant influence of prior environmental conditions on the timing and intensity of Eucalypt flowering^8,28^, we also derived time-lagged differences of these variables at 1, 3, 6, 9, and 12 month time lags. Also, we added distance from the home roost as a predictor variable to help model spatial autocorrelation of the foraging data not addressed by data aggregation and modeling techniques such as bagging and spatial cross validation (discussed below). For a complete list of variables explored in the model, see Table 3.

### Foraging data

**Figure 12:**
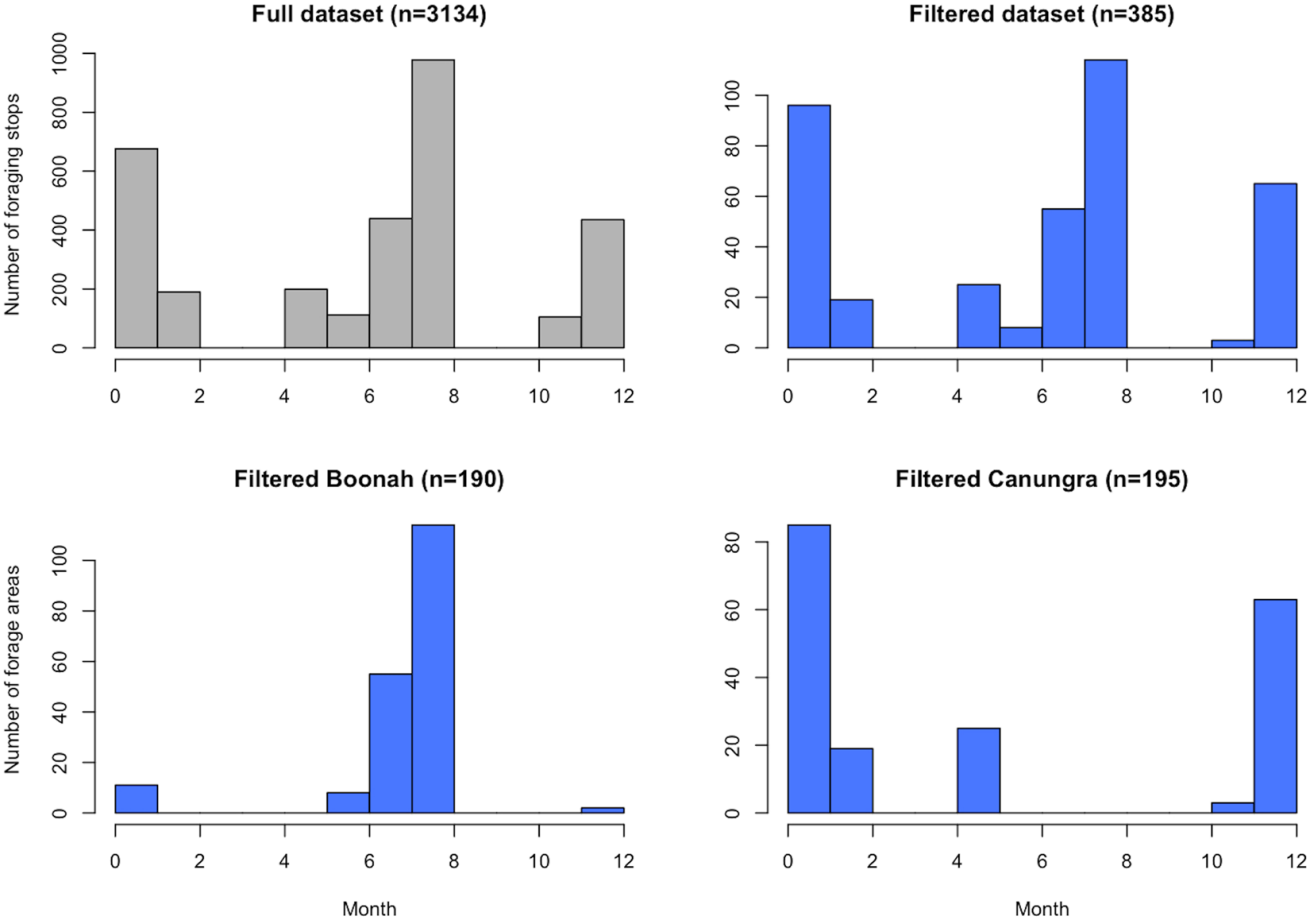
Histograms plotting the number of foraging stops of *Pteropus poliocephalus* recorded at roosts in Boonah and Canungra in southeastern Queensland. The month is plotted on the y-axis and the x-axis shows the number of individual foraging stops in the top panel, and the number of aggregated foraging areas in the bottom panel. The full unfiltered data set is shown in grey at the top left. The filtered data (clustered foraging stops, urban points removed, aggregated to resolution of environmental data) is shown in blue. Data were not retrieved from individual bats in Mar/Apr and Sep/Oct (see top right). Foraging activity tends to occur in the winter around the Boonah roost, and in the summer around the Canungra roost. For a detailed description of the movement data, see Westcott *et al*.^4^.

**Figure 13:**
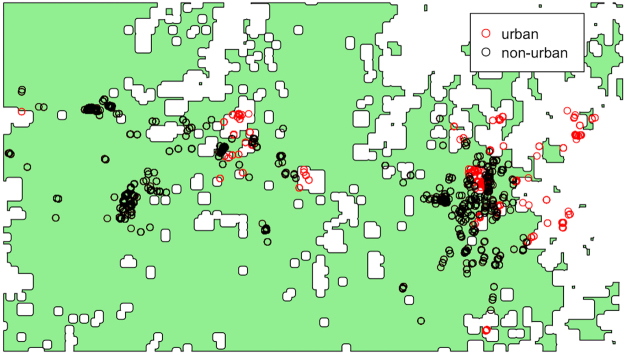
The spatial distribution of *Pteropus poliocephalus* foraging areas (aggregated foraging stops; see Methods) plotted around their home roosts in Boonah and Canungra in southeastern Queensland. The area shaded green represents non-urban areas and the white areas represent urban and peri-urban areas. Observed foraging areas that are found within non-urban areas are plotted as black circles and the red circles indicate those in urban and peri-urban areas.

Data on bat movement was collected as part of the Nation Flying-fox Monitoring Program^4^. Specifically, Westcott *et al*.^4^ fitted 37 GHFF individuals (15 females and 22 males) with tracking devices enabled with GPS and accelerometers from June 2013–Feb 2015. We then aggregated the raw data on two levels: first, by clustering movement data into foraging stops, and second, by counting the number of points aggregated to the spatial and temporal resolution of the environmental data. Fine-scale movement data was filtered to identify times and locations of significant foraging activity. We considered bats to be in a ‘stopped’ state when the speed of the device was recorded as <2 ms^−1^. We defined a successful foraging event (hereafter referred as a foraging ‘stop’) as an area where a group of stop points was within 10 meters of one another. Accordingly, we produced a dataset of foraging ‘stops’ by clustering points within 10 m and taking the centroid, rendering 2997 points with a spatial distribution shown in Fig. 1. Although the data have an even distribution across gender (female = 1440, male = 1557), season (summer = 1318, winter = 1679), and the two home roosts in our study area (Boonah = 1590, Canungra = 1407), movement data was not retrieved from any bats in Mar/Apr and Sep/Oct, and the bats exhibit a preference for foraging near Canungra in the summer and Boonah in the winter (Fig. 12). For more detailed specifications of the GPS technology developed for the tracking collars, see Sommer *et al*.^59^, and for additional description of their implementation to collect movement data, see Westcott *et al*.^4^. To remove the potential confounding effect of generalist foraging behavior compared with foraging on nectar-based resources in Eucalypt forests, we removed points within 500 m of urban and peri-urban land use types as this was expected to contain high proportions of non-native fruiting trees, leaving 2416 foraging stops that are likely driven by Eucalypt phenology (Fig. 13). To attain counts of foraging stops per unit area and time, we aggregated the data to the temporal and spatial resolution of the environmental data. We aggregated foraging stops temporally by assigning them to the nearest 8-day interval of the environmental data, and then spatially by counting the number of foraging stops within each grid cell, resulting in aggregated counts for 385 unique locations and times.

To account for sampling bias and provide information on the environments of foraging sites that are available, but not used, by our 37 individual bats, we implemented a use-availability sampling design^60,61^. Specifically, we compiled a data set of ‘available’ foraging sites, for which the response variable (number of foraging stops) was set to zero, by drawing a large number of randomly stratified points within the maximum observed foraging distance of each roost (46 km for Boonah and 30 km for Canungra; see Fig. 1), for all dates with observed foraging activity. We adjusted the total number of points so that an ‘available’ foraging site was sampled once per 5 km^2^ within each of the 25 unique dates (approximately 19000 points total). This sampling design provides a thorough representation of the environmental space at available foraging sites within the foraging radius of the home roosts at times when individuals were roosting there^60^; however, it results in imbalanced data classes that can lead to underestimation of fitted values and hinder model assessment^62^. We assumed that both ‘used’ and ‘available’ foraging areas are equally accessible based on observed foraging radii. So, before modeling, we applied a correction that balances the weight of the two data classes. Following King and Zeng^62^, we weighted ‘used’ samples as ${w}_{1}=\tau /\bar{y}$, and ‘available’ samples as ${w}_{0}=(1-\tau )/(1-\bar{y})$. Where *τ* is set to 0.5, and $\bar{y}$ is the frequency of ‘used’ foraging areas in the data.

### Modeling

To estimate spatiotemporal foraging intensity, we explored models using boosted regression trees (BRT) with a Poisson error distribution and a log link function. This gives a functional approximation for the expected density of foraging stops within a grid cell at a given time relative to available foraging areas within the foraging radii around our study roosts. We used BRT because regression trees provide an additive model structure that can fit a flexible non-monotonic response that ignores weak covariates^63,64^. The BRT algorithm also reduces the impact of autocorrelation that may result from spatial clustering in the data via ‘bagging’, where models at each iteration of the algorithm are built with a randomly selected proportional subset of the data^65^. BRT is especially useful when modeling environmental patterns associated with flying-fox foraging areas because they forage on a wide range of Eucalypt species and forest types, each with a potentially unique pattern of climatically driven flowering. The flexible response fitted by BRT can accommodate the complex relationship between Eucalypt phenology and bat foraging behavior within a framework similar to classic Poisson regression. Moreover, recent literature has elucidated the relative proportionality among Poisson regression, presence-background regression, and inhomogenous Poisson point processes when modeling the intensity of a point process^66–68^.

**Figure 14:**
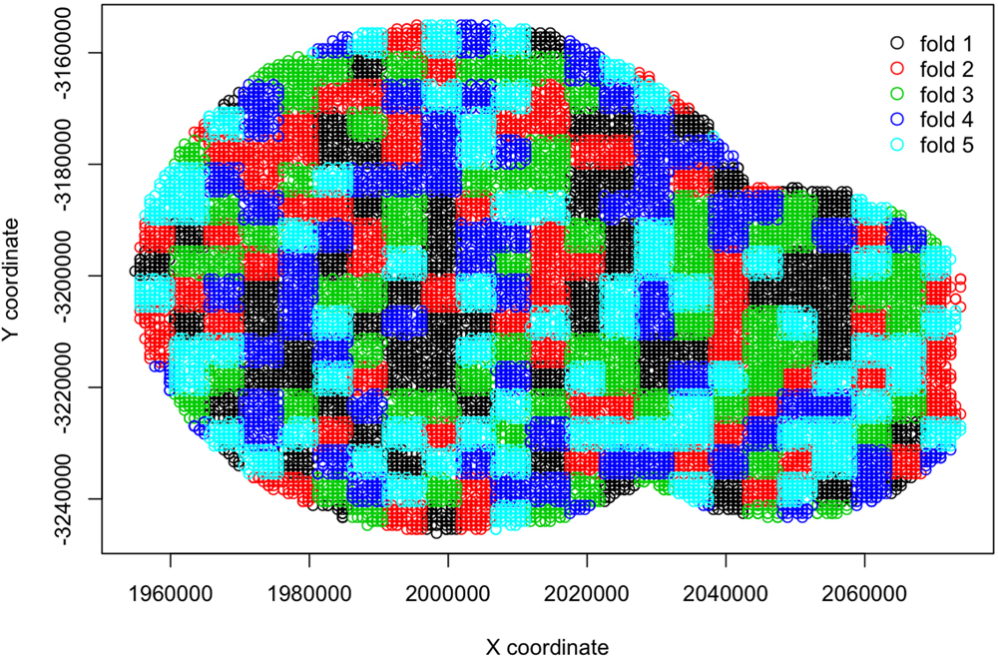
The spatial cross validation design for testing the boosted regression tree models assigns points within the maximum observed foraging radius around the two study roosts in southeastern Queensland (46 km for Boonah and 30 km for Canungra) to one of five randomly assigned 5 km grid cells. Each color indicates membership to one of the five folds.

To identify an optimal model, we followed the recommendations of Elith *et al*.^64^, where we explored the performance of models built with various combinations of meta-parameters such as tree complexity (*tc* = 5, 7, 9), learning rate (*lr* = 0.005, 0.001, 0.0005), and bagging fraction (*bf* = 0.5, 0.6, 0.7). Out of the 27 combinations, the optimal meta-parameter set was determined based on performance on independent test data within a 5-fold spatial cross validation structure. Cross validation folds were assigned using a 5 km spatial grid placed over the study area (defined by maximum foraging radii). Points were assigned to one of the 5 folds based on random assignment of the grid cells into one of the 5 folds (similar to Renner and Warton^67^; Fig. 14). To ensure reliable metrics in cross validation tests, we selected a random seed that ensured even proportion of used and available foraging areas across the 5 folds. We tested model generality by calculating the mean Poisson deviance and mean squared error (MSE) among the 5 folds. The final selected model was refitted with all data using the meta-parameter combination that returned the lowest mean deviance and MSE, while fitting >1000 trees (as suggested by^64^). Then we re-fitted the final model with all the data to determine the optimal number of trees on the full data set.

To describe the fitted function, we selected 12 influential variables (shown in bold in Table 3) and constructed partial dependence plots by predicting the model over the range of the variable in question while holding all other variables in the model constant. We also explored the influence of interactions among environmental variables and plotted the two most influential interactions. To assess the spatiotemporal pattern of GHFF foraging intensity across the study area, we projected the final model onto the spatial data at 8-day intervals from 2006–2015. We then aggregated predictions to monthly summaries, by taking the mean at each grid cell within each month. We summarized temporal trends in two ways. First, we plotted a time series of violin plots that show the distribution of all cell values for the monthly spatial predictions. Second, we plotted a univariate time series that gives the quartile variation coefficient (QVC; a measure of relative dispersion suited to non-normal count data). Here, high values of the QVC correspond to spatial predictions that produce high values of foraging intensity surrounded by comparatively low values (indicative of high-quality nectar production events), and low values correspond to those with a more homogeneous distribution of low or mid-range values (indicative of poor foraging conditions and dispersed foraging behavior). In this manner, the QVC can be interpreted as a measure of spatial resource concentration.

To assess temporal trends in spatial resource concentration, we plotted model values by year and month and validated them with independent data of population counts. Specifically, we calculated time series of QVC values for spatial predictions within the maximum foraging radius of Canungra (30 km) and compared them to the median value of monthly population counts observed at Canungra from 2008–2014 using a Pearson’s product moment test. We focus on the Canungra roost here because over this time period, consistent monthly population counts of *Pteropus poliocephalus* were performed by the Queensland Department of Environment and Heritage Protection as part of the Southeast Queensland Flying-fox Monitoring Program. Population counts were also performed at the Boonah roost from 2011–2014, however, this roost was subjected to repeated dispersal attempts during this time, leading up to its removal in 2014. We also explored the connection between QVC across the whole study area from 2006–2015, and a broad climate indicator, the Southern Oscillation Index (SOI^69^. In this case, we calculated the cross-correlation function between the temporal summary of the model and the 3-month moving average of monthly SOI values at time-lags of up to 18 months.

To explore hypotheses on how eucalypt phenology and observed foraging activity are linked to Hendra virus dynamics, we gathered data on Hendra virus prevalence and spillover occurrence within our study area and plotted seasonal trends with our model of nectar-based foraging resources. We acquired data on Hendra virus prevalence in fruit bats from the field sampling study conducted by Field *et al*.^26^. As described by Field *et al*.^26^ prevalence values are calculated from pooled urine samples collected under the roost. Although both GHFF and BFF were present at this roost at the time of sampling, Hendra virus excretion is primarily attributed to BFF^15^. Within our study area, Hendra virus prevalence data was only available for the Boonah roosting site. The times and locations of Hendra virus spillover events to horses came from the Queensland Government’s public database of documented Hendra virus infection in horses^27^. To summarize seasonal patterns in the data, we fit Generalized Additive Models (GAMs) with cubic cyclic spline smoothing terms to monthly values of spatial resource concentration (approximated with the Quartile Variation Coefficient (QVC)) and Hendra virus prevalence using Gaussian and Quasi-Binomial link functions respectively. The spline terms used integer values corresponding to each month as the covariate. We calculated the total number of monthly spillover events which occurred within three distance thresholds (25, 50, and 100 km) of the two study roost sites and plotted all three data sources together. All data processing and modeling was performed in the R statistical language^70^ using the mgcv, gbm, dismo, raster, and spatstat packages^71–75^. Data and R code can be found in the online supplementary information.

## Electronic supplementary material

Supplementary R code
