# Supplementary material for: Environmental drivers of spatiotemporal foraging intensity in fruit bats and implications for Hendra virus ecology
Source: Sci Rep. 2018 Jun 22;8:9555. doi: 10.1038/s41598-018-27859-3 (PMC6015053; doi:10.1038/s41598-018-27859-3)
Supplement: Supplementary file 1 — Supplementary R code [file 41598_2018_27859_MOESM1_ESM.pdf]

Environmental drivers of spatiotemporal foraging intensity in fruit bats and implications for Hendra virus ecology

John R. Giles, Peggy Eby, Hazel Parry, Alison J. Peel, Raina K. Plowright, David Westcott, and Hamish McCallum

R code for boosted regression tree analysis

```
library(raster)
library(dismo)
library(SDMTools)
library(Metrics)
library(foreach)
library(parallel)
library(doParallel)

# read data
path <- 'data/foraging_stop_data.csv' # file path to foraging_stop_data.csv
d <- read.csv(path)

# Albers equal area coordinate projection
albers <- "+proj=aea +lat_1=-18 +lat_2=-36 +lat_0=0 +lon_0=132 +x_0=0 +y_0=0
+ellps=GRS80 +towgs84=0,0,0,0,0,0,0 +units=m +no_defs"

# weight/offset
ybar <- sum(d$pres)/nrow(d) # probability of presence in dataset
tau <- 0.5 # assumed probability of presence
w1 <- tau/ybar
w0 <- (1-tau)/(1-ybar)
d$weight <- round(w1*d$pres + w0*(1-d$pres), 2)

# add gridded groups
dpts <- SpatialPointsDataFrame(cbind(d$coords.x1, d$coords.x2), d)
cols <- 23
rows <- 18 # 5km cells
par(mfrow=c(1,1), mar=c(4,4,2,2))
r <- raster(ncol=cols,
            nrow=rows,
            ext=extent(dpts),
            crs=CRS(albers),
            vals=1:(rows*cols))
dpts$gridcell <- raster::extract(r, dpts)
print(ncells <- length(unique(dpts$gridcell))/5) # grid cells per fold

# aggregate data into 5 random groups
m <- matrix(nrow=0, ncol=2)
try <- 1000
for (i in 1:try) {
     set.seed(i)
     ggall <- unique(dpts$gridcell) # vector of all gridgroups
```

```r
    gg1 <- sample(ggall, ncells)
    dpts1 <- dpts[dpts$gridcell %in% gg1,]
    dpts1$gridgroup <- 1
    ggall <- ggall[!(ggall %in% gg1)] # remove gg1 sample from ggall
    gg2 <- sample(ggall, ncells)
    dpts2 <- dpts[dpts$gridcell %in% gg2,]
    dpts2$gridgroup <- 2
    ggall <- ggall[!(ggall %in% gg2)]
    gg3 <- sample(ggall, ncells)
    dpts3 <- dpts[dpts$gridcell %in% gg3,]
    dpts3$gridgroup <- 3
    gg4 <- ggall[!(ggall %in% gg3)]
    gg4 <- sample(ggall, ncells)
    dpts4 <- dpts[dpts$gridcell %in% gg4,]
    dpts4$gridgroup <- 4
    gg5 <- ggall[!(ggall %in% gg4)] # grid cells remaining in ggall
    dpts5 <- dpts[dpts$gridcell %in% gg5,]
    dpts5$gridgroup <- 5
    print(i)

    # use seed which has lowest variance amoung grid groups
    print(s <- round(var(c(sum(dpts1$pres),
                           sum(dpts2$pres),
                           sum(dpts3$pres),
                           sum(dpts4$pres))),1))
    m <- rbind(m, c(i, s))
}

# use seed with data most evenly distributed across grid groups
m <- m[order(m[,2]),]
set.seed(m[1,1])

# assign to grid groups
ggall <- unique(dpts$gridcell) # vector of all gridgroups
gg1 <- sample(ggall, ncells)
dpts1 <- dpts[dpts$gridcell %in% gg1,]
dpts1$gridgroup <- 1
ggall <- ggall[!(ggall %in% gg1)] # remove gg1 sample from ggall
gg2 <- sample(ggall, ncells)
dpts2 <- dpts[dpts$gridcell %in% gg2,]
dpts2$gridgroup <- 2
ggall <- ggall[!(ggall %in% gg2)]
gg3 <- sample(ggall, ncells)
dpts3 <- dpts[dpts$gridcell %in% gg3,]
dpts3$gridgroup <- 3
ggall <- ggall[!(ggall %in% gg3)]
gg4 <- sample(ggall, ncells)
dpts4 <- dpts[dpts$gridcell %in% gg4,]
dpts4$gridgroup <- 4
gg5 <- ggall[!(ggall %in% gg4)] # grid cells remaining in ggall
dpts5 <- dpts[dpts$gridcell %in% gg5,]
dpts5$gridgroup <- 5
```

```
sum(dpts1$pres)
sum(dpts2$pres)
sum(dpts3$pres)
sum(dpts4$pres)
sum(dpts5$pres)

# recombine spatialpointsdataframes
d <- rbind(as.data.frame(dpts1),
           as.data.frame(dpts2),
           as.data.frame(dpts3),
           as.data.frame(dpts4),
           as.data.frame(dpts5))
d <- d[,-c((ncol(d)-1),ncol(d))]

plot(d$coords.x1, d$coords.x2, pch=1, col=d$gridgroup)
lines(rasterToPolygons(r), lwd=2)

# build boosted regression tree models with all combinations of meta-parameters
# and cross validate using the random grid groups as folds
LR <- c(0.005, 0.001, 0.0005) # learning rate
TC <- c(5,7,9) # tree count
BF <- c(0.5,0.6,0.7) # bagging fraction

results.all <- list()
for(g in 1:max(d$gridgroup)) {

     # define gridded CV subsets
     print(paste("Withholding fold number ", g, sep=" "), quote=F)
     d.train <- subset(d, gridgroup != g )
     d.test <- subset(d, gridgroup == g)

     df <- data.frame()
     for (i in TC){
          for (j in LR){
               for (k in BF) {

                    print(paste('TC = ', i, ', LR = ', j, ', and BF = ', k, sep=''),
                          quote=F)

                    BRT1 <- gbm.step(data=d.train,
                                     gbm.x=c(4,8:18), #c(4,8:18,24,30:63)
                                     gbm.y=1,
                                     family='poisson',
                                     tree.complexity=i,
                                     learning.rate=j,
                                     bag.fraction=k,
                                     n.folds=5,
                                     site.weights=d.train$weight,
                                     max.trees=10000,
                                     verbose=F)

                    if(is.null(BRT1)==T){
                         tmp <- data.frame(TC=i,
```

```r
                                          LR=j,
                                          BF=k,
                                          n.trees=NA,
                                          deviance=NA,
                                          MSE=NA,
                                          R2=NA)
                    } else {
                         preds <- predict(BRT1,
                                          d.test,
                                          n.trees=BRT1$gbm.call$best.trees,
                                          type='response')

                         dev <- calc.deviance(obs=d.test$count,
                                              pred=preds,
                                              family='poisson',
                                              calc.mean=T)

                         MSE <- mse(d.test$count, preds)
                         R2 <- cor(d.test$count, preds)^2

                         # add results to list
                         name <- paste('TC', i, 'LR', j, 'BF', k, sep='')
                         tmp <- data.frame(TC=i,
                                           LR=j,
                                           BF=k,
                                           n.trees=BRT1$gbm.call$best.trees,
                                           deviance=dev,
                                           MSE=MSE,
                                           R2=R2)
                    }

                    df <- rbind(df, tmp)
               }
          }
     }
     results.all[[g]] <- df
}

df <- results.all[[1]][,colnames(results.all[[1]]) %in% c('TC', 'LR', 'BF')]

# get summaries of deviance and MSE
for (i in c('deviance','MSE')){
     dv <- as.data.frame(cbind(results.all[[1]][,i],
                               results.all[[2]][,i],
                               results.all[[3]][,i],
                               results.all[[4]][,i],
                               results.all[[5]][,i]))
     dv <- as.data.frame(cbind(round(apply(dv,
                                          FUN=mean,
                                          MARGIN=1), 3),
                               round(apply(dv,
                                          FUN=sd,
                                          MARGIN=1), 2)
```

```r
    ))
    names(dv) <- c(paste(i, '.mean', sep=''),
                   paste(i, '.se', sep=''))
    df <- cbind(df, dv)
}

# confirm n.trees was >1000 for all cross validations
dv <- as.data.frame(cbind(results.all[[1]][,'n.trees'],
                          results.all[[2]][,'n.trees'],
                          results.all[[3]][,'n.trees'],
                          results.all[[4]][,'n.trees'],
                          results.all[[5]][,'n.trees']))

df$n.trees <- apply(dv,
                    FUN=function(x) min(x),
                    MARGIN=1)
df$min.trees <- apply(dv,
                      FUN=function(x) min(x) > 1000,
                      MARGIN=1)

df2 <- subset(df, n.trees > 1000)
df2 <- df2[order(df2$deviance.mean),]
best.pars <- df2[1,]

# Re=run gbm.step with best parameters to get optimal number of trees
BRT1 <- gbm.step(data=d,
                 gbm.x=c(4,8:18,24,30:63),
                 gbm.y=1,
                 family='poisson',
                 tree.complexity=best.pars$TC,
                 learning.rate=best.pars$LR,
                 bag.fraction=best.pars$BF,
                 n.folds=5,
                 site.weights=d$weight)

preds <- predict(BRT1,
                 d,
                 n.trees=BRT1$gbm.call$best.trees,
                 type='response')

calc.deviance(obs=d$count,
              pred=preds,
              family='poisson',
              calc.mean=T)

# Summarize Poisson model performance
par(mfrow=c(1,2), mar=c(4,4,2,2))

plot(BRT1$fitted, BRT1$residuals,
     ylab='Model residuals',
     xlab="Fitted values")
abline(h=0, lty=2, col='grey50')
sp <- smooth.spline(BRT1$fitted, BRT1$residuals, spar=2.09)
```

```
lines(sp, col='red', cex=1.2)

plot(d$count, BRT1$fitted,
     ylim=c(0,max(d$count)),
     ylab='Fitted values',
     xlab="Data")
lines(x=seq(-5, max(d$count)+5, 1),
      y=seq(-5, max(d$count)+5, 1),
      lty=2,
      col='grey50')
sp <- smooth.spline(BRT1$fitted, d$count, spar=2)
lines(sp, col='red', cex=1.2)

R2 <- cor(d$count, BRT1$fitted)^2
lab <- bquote(italic(.('R'))^2 == .(round(R2, 2.1)))
text(6, 35, label=lab)

# Influential variables
par(mfrow=c(1,1))
barplot(summary(BRT1)[1:24,])
```
